# Assessing behaviour of osteoblastic cells in dynamic culture conditions using titanium-doped phosphate glass microcarriers

**DOI:** 10.1177/2041731419825772

**Published:** 2019-02-08

**Authors:** David De Silva Thompson, Carlotta Peticone, Iva Burova, Rebecca J Shipley, Jonathan C Knowles, Hae-Won Kim, Martina Micheletti, Ivan B Wall

**Affiliations:** 1 Department of Biochemical Engineering, University College London, London, UK; 2 Department of Mechanical Engineering, University College London, London, UK; 3 Division of Biomaterials and Tissue Engineering, Eastman Dental Institute, University College London, London, UK; 4 The Discoveries Centre for Regenerative and Precision Medicine, University College London, London, UK; 5 Department of Nanobiomedical Science and BK21 PLUS NBM Global Research Center for Regenerative Medicine, Dankook University, Cheonan, Republic of Korea; 6 UCL Eastman-Korea Dental Medicine Innovation Centre, Dankook University, Cheonan, Republic of Korea; 7 Institute of Tissue Regeneration Engineering (ITREN), Dankook University, Cheonan, Republic of Korea; 8 Aston Medical Research Institute and School of Life & Health Sciences, Aston University, Birmingham, UK

**Keywords:** Bone, tissue engineering, phosphate glass, Froude number

## Abstract

Tissue engineering is a promising approach for bone regeneration; yet challenges remain that limit successful translation to patients. It is necessary to understand how real-world manufacturing processes will affect the constituent cells and biomaterials that are needed to create engineered bone. Bioactive phosphate glasses processed into microspheres are an attractive platform for expanding bone-forming cells and also for driving their osteogenic differentiation and maturation. The aim of this study was to assess whether Ti-doped phosphate glass microspheres could support osteoblastic cell responses in dynamic cell culture environments. Dynamic culture conditions were achieved using microwell studies under orbital agitation. Dimensionless parameters such as the Froude number were used to inform the choice of agitation speeds, and the impact on cell proliferation and microunit formation was quantified. We found that phosphate glass microspheres doped with titanium dioxide at both 5 and 7 mol% provided a suitable biomaterial platform for effective culture of MG63 osteoblastic cells and was not cytotoxic. Dynamic culture conditions supported expansion of MG63 cells and both 150 and 300 rpm orbital shake resulted in higher cell yield than static cultures at the end of the culture (day 13). The Froude number analysis provided insight into how the microunit size could be manipulated to enable an appropriate agitation speed to be used, while ensuring buoyancy of the microunits. These small-scale experiments and analyses provide understanding of the impact of fluid flow on cell expansion that will have increasing importance when scaling up to process technologies that can deliver clinical quantities of cell-microsphere units. Such knowledge will enable future engineering of living bone-like material using processing systems such as bioreactors that use mixing and agitation for nutrient transfer, therefore introducing cells to dynamic culture conditions.

## Introduction

Bioactive glass is a promising material for bone tissue engineering. Human osteoblasts can be cultured on 45S5 BioGlass^®^ and sol-gel derived 58S, where they produce collagenous extracellular matrix (ECM) and ultimately bone nodules without the need for supplements or growth factors.^1,2^ When cultured on silicate-based glass materials, at least threefold increase in osteoblast proliferation can be observed.^3^ Previous work showed that human MG63 osteoblastic cells can be cultured and expanded on phosphate glass discs,^4,5^ indicating that this material is also cytocompatible. Phosphate glasses are therefore a candidate for bone tissue formation. The materials’ ability to be completely soluble in aqueous media^6^ means it can potentially be implanted and remodelled in vivo.

The phosphate glass composition makes it possible to dope ions into the glass by replacing Na_2_O with, for example, TiO_2_. TiO_2_ improves the stability of the phosphate glass due to the high field strength observed with its oxide. The efficacy of titanium phosphate glass microspheres (Ti-PGMs) to provide a platform for growth of MG63 cells is understood for static cell culture.^7^ However, the application of this material must be extended to dynamic cell culture if it is to be used in tissue engineering at a commercially and clinically relevant scale.

Biomaterial scaffolds processed into microcarriers have been studied extensively^8–10^ and carry many benefits over conventional ‘static’ scaffolds. For example, they offer flexibility for filling defects of different shapes and sizes. In theory, larger defects can be accommodated by increasing the amount of microcarriers and cells but use the same bioreactor culture to scale up quantities. In fact, microcarriers are routinely used to scale up cell culture of adherent cells, but predominantly use plastic microcarriers,^11^ from which the cells have to be detached and separated. However, for bone tissue engineering purposes, the material also needs to be implantable and capable of supporting biological enhancements in vivo.^12^

It is important to understand the operating ranges required for microcarrier-based cell culture, in order to achieve the scale up that microcarriers aim to enable. Currently, less than 2% of relevant publications have focused on the use of shaken cell culture platforms (flasks or microwells) and therefore, limitations exist when trying to scale up shaken platforms to larger volumes and even to alternative bioreactors such as stirred vessels, especially for two phase culture systems.^13^ Shaken platforms are beneficial in that they can be designed at microwell scale into multi-well formats providing the opportunity to examine an array of conditions. An additional advantage with shaken flasks when compared to other stirred bioreactor systems is the reduced formation of damaging bubble bursts due to air sparging.^14^ Operating parameters including the flask’s shape and size, the orbital agitation speed and diameter, and properties of liquid and particles within the flask can all impact on bioprocessing factors such as oxygen transfer, cell growth and cell viability.^13,15^ Progress has been made in understanding how fluid dynamics in orbitally shaken vessels are responsible for the suspension of microspheres. There is a trade-off between increasing flow rates to suspend particles, improve nutrient transfer and provide fluid shear stimulation, versus causing cell damage if flows become too high; mathematical modelling is a useful tool to elucidate this balance.

Previous work has used dimensional analysis to explore the role of energetics and metabolism in bioreactors. For example, power consumption and volumetric mass transfer coefficients have been determined through dimensionless analysis to establish ‘macro-parameters’ that provide an overall assessment of the bioreactor, rather than looking at flow dynamics at varying operating conditions^16–18^; however, these parameters do not account for the fluid flow environment. Here, we introduce the Froude number (Fr) (equation (1)), which represents the ratio of flow inertia forces compared to gravity and describes the resistance of a submerged object moving through the fluid. We define Fr as the ratio between the characteristic flow velocity (v) and the wave propagation velocity ($\sqrt{gl}$) where *g* is gravitational acceleration and *l* is the characteristic length scale^19^

(1) $$Fr=\frac{v}{\sqrt{gl}}$$

In Tissot et al.,^20^ the authors estimated the characteristic velocity based on the average of the orbital and inner cylinder diameters ($d_{o}$ and $d_{i}$, respectively), while the inner cylinder diameter was taken as the characteristic length scale to give

(2) $$Fr=\left(\frac{2\pi N\left(d_{i}+d_{0}\right)/2}{\sqrt{gd_{i}}}\right)$$

where N is the shaker rotation speed. Equation (2) is used widely in the work of Ducci and Weheliye^15,21^ to describe the flow in orbital bioreactors, has been validated against particle image velocimetry (PIV) measurements and is the basis of the approach taken here.

**Figure 1.**
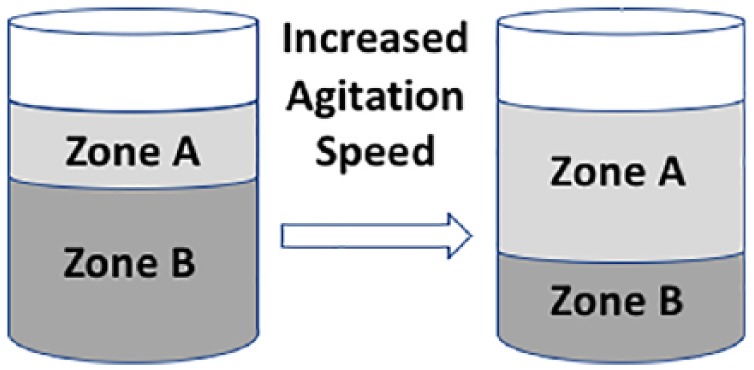
Mixing zones in a cylindrical vessel. The image highlights the change in height of mixing zone (Zone A) when fluid in a cylindrical vessel is subjected to increased agitation.

Weheliye et al.^15^ went on to consider how varying the shaker rotation speed can impact on fluid mechanics in the bioreactor, in particular it’s effects on mixing of nutrients and culture products. Ensuring effective mixing is essential, as the presence of spatial gradients in culture produces heterogeneous products, and this necessitates understanding the types of mixing regimes that emerge within a well. For low agitation speeds, counter rotating toroidal vortices form (Figure 1). These vortices are present only in the upper part of the fluid in the well, which we refer to as Zone A. In the region below these vortices, Zone B, there is a relatively stagnant region due to lack of exposure to these vortices. These regions are also referred to as the convection (A) and diffusion (B), due to the dominant transport mechanism associated with each. Upon an increase in agitation speed, the vortices extend to the bottom of the vessel with their intensity increasing in magnitude, hence incorporating both zones within the mixing system. This distribution of different zones within the vessel was validated using PIV measurements carried out at a range of shaker rotations speeds. At even higher agitation rates (and hence also higher *Fr*), there is a shift to out-of-phase flow, where the movement of the fluid is not in sync with that of the shaking platform.^15^

This out-of-phase flow regime maximizes mixing and therefore is optimal in terms of generating a homogeneous nutrient distribution, and also homogeneous distribution of suspended microspheres. Generation of out-of-phase flow was explored using a combination of theory and experiments in Tissot et al.,^20^ who concluded that it may be predicted and controlled based on the Froude number, *Fr*, the non-dimensional fluid height (*h*/*d_i_*, where *h* is the fluid height) and the non-dimensional orbital diameter (*d_o_*/*d_i_*). Essentially, if $h/d_{i}\leq \surd d_{o}/d_{i}$, the vortices extend to the base of the cylindrical vessel before phase transition occurs and the Froude number can be estimated by

(3) $$Fr=\frac{1}{a_{0}}\frac{\Delta h}{d_{i}}$$

where D*h* Δh is the free surface height and $a_{0}$ is the constant of proportionality ($a_{0}=1.4$ for water). If, instead, $h/d_{i}>\surd d_{o}/d_{i}$, the transition to out-of-phase flow occurs without the vortex reaching the bottom of the vessel and a diffusion zone will be present. In this case, the transitional velocity for a given configuration depends only on the inner cylinder diameter via

(4) $$Fr_{d_{i}}=\frac{1}{a_{0}}$$

where $Fr_{d_{i}}$ is the Froude number based on the cylinder inner diameter. In each scenario, for a given vessel geometry, equations (3) and (4) enable the minimum agitation velocity (and hence Froude number) to be chosen to promote mixing.

The aim of this study was to assess whether Ti-PGMs can be used as a substrate for cell culture under dynamic culture conditions. The experiments were carried out using MG63 cells because they are a well-established tool for biocompatibility studies and their robustness enables bioprocess boundaries to be explored.^7,8,22–24^ Based on previous observations of the positive effect of fluid flow shear stress under laminar flow conditions,^25^ it was hypothesized that dynamic agitation conditions would stimulate MG63 cell proliferation due to the associated fluid flow shear stress. Agitation rates were chosen using the arguments presented above, based on the exact geometries of the wells used. Furthermore, we sought to examine whether any dose-dependent improvement in cell responses to TiO_2_ would continue beyond 5 mol%, and therefore, a concentration of 7 mol% was also tested. No higher concentrations were assessed due to increase in density and stability reported with glasses containing TiO_2_ above 10 mol%.^4^ Using the Froude analysis to determine the appropriate mixing regimes when using TiO_2_ will define the operating parameters required to use this biomaterial at commercially relevant scales.

## Methods

### Formulation/preparation of Ti-PGMs

The phosphate-based glass was manufactured according to techniques described in Abou Neel and Knowles,^5^ where stoichiometric quantities of the following precursor were mixed in a Seward Stomacher^®^ 400 Circulator (Wolf Laboratories, York, UK) at 200 rpm for 1 min (unmodified purities of >99%, obtained from VWR-BDH, Poole, UK): phosphorus pentoxide, (P_2_O_5_), calcium carbonate (CaCO_3_), sodium dihydrogen orthophosphate (NaH_2_PO_4_) and titanium dioxide (TiO_2_) (Table 1). The precursor mix was consequently poured into a Pt/10% Rh type 71040 crucible (Johnson Matthey, Royston, UK). The process initiates with the removal of CO_2_ and H_2_O by preheating the composition at 700°C and then melting the resulting mixture at a composition-specific temperature listed below. After melting glasses under the conditions indicated above, the glasses were quenched by pouring onto a steel plate at room temperature and allowing to cool overnight.

**Table 1.** Glass compositions.

Glass composition	Composition breakdown (mol.%)				Processing temperature
	Calcium oxide	Sodium oxide	Phosphorus pentoxide	Titanium dioxide	Melting temperature (°C)/time (h)
**P50Ca30Na20Ti0 (0 mol%)**	30	20	50	0	1100/1
**P50Ca30Na15Ti5 (5 mol%)**	30	15	50	5	1300/3
**P50Ca30Na13Ti7 (7 mol%)**	30	13	50	7	1350/5.5

Modified from Abou Neel et al.^4^

After a solid structure is formed, the quenched glass was fragmented into microspheres using methods described in Lakhkar et al.^8^ To separate the required fraction of microspheres for use during tissue culture, the particles were sieved down to 63–106 µm diameter. This microsphere size range is hypothesized to be optimal; previous studies indicate that smaller particles are associated with ineffective spheroidization after fragmentation, while larger fractions having a shorter residence time during the spheroidization step, generating irregular structures unsuitable for cell attachment and culture.^8^ The glass microspheres generated after the sieving stage do not automatically possess a spherical structure required for ideal tissue adherence; therefore, simplistic spheroidization apparatus was used to generate the required three-dimensional spherical structure.^8^ The spheroidization set up included the following components: (1) blow torch, (2) feed and (3) collectors. The blow torch assembly is composed of a Rothenberger Superfire 2 gas torch (Rothenberger Werkzeuge GmbH, Kelkheim, Germany), fitted to a 453g MAP-PLUS gas canister (Todays Tools, UK), a substitute for the standard MAPP gas (methylacetylene-propadiene propane) which generates a high flame temperature of 2925°C in the presence of oxygen. The feed consists of an aluminium trough (200 × 20 × 30 mm) with the outlet edge located ~10 mm above the torch at an angle to the horizontal. A DC motor (15,800 rpm, 4.5–15 V, 35.8 mm diameter; RS Components, Corby, UK) is attached to the other end of the trough and is connected to a programmable power supply (RS Components). A metal screw attached to the axle of the motor provides an offset, generating the vibrations required to propagate the microspheres through the trough, before entering the flame. The collecting section is composed of four glass trays (275 × 150 × 60 mm) in an arrangement which sees the longer edges of each tray in contact with each other. The first tray is placed directly under the flame as to collect the particles that do not fall into the flame. Each consequent tray is placed in the axis of the flame as to collect any beads that enter its path. As the microparticles pass through the flame, they undergo a spheroidization due to the surface tension forces and fall into the four trays placed below. Upon spheroidization, the resulting microspheres were imaged using light microscopy to verify their spherical geometry.

### Preparation of MG63 cells

A human osteoblast-derived cell line (MG63) was used to assess the biological response to titanium-doped biomaterials. These cells were obtained from in-house stocks (P3P9) from the Biochemical Engineering Department, University College London. Cells were first cultured in a 75 cm^2^ T-Flask at a seeding density of 10,000 cells/cm^2^ using Dulbecco’s Modified Eagle’s Medium (DMEM) (1 g/L glucose), supplemented with foetal bovine serum (10%) and antibiotic–antimycotic solution (1%) (all reagents acquired from Gibco^®^, Life Technologies Ltd., Paisley, UK, unless specified otherwise) at 37°C and 5% CO_2_. Passages were carried out when ~80% confluency was reached, established by visual observation through phase contrast microscopy. Cell was then washed, trypsinized and quantified using a Neubauer haemocytometer and viability determined using Trypan blue dye exclusion method.^26^

### MG63 cell culture on Ti-PGMs under static and dynamic conditions

Static and dynamic culture of the MG63 cells of Ti-PGMs was carried out in ultra-low attachment 96-well plates (Corning^®^ Costar^®^, Sigma-Aldrich, UK) to ensure that undesired cell attachment did not occur. A pre-calculated quantity of microspheres (5 mg/well (±0.1 mg)) of each titanium concentration was inserted into wells of a 96-well plate in order to cover the surface area of the well in a monolayer. These microspheres were ultraviolet (UV) sterilized for 1 h and 40 min using a high-intensity Blak-Ray B-100SP lamp (UVP, Cambridge, UK), then removed and equilibrated with 135 µL of pre-warmed media to avoid direct contact of the cells with the dry surface of the microspheres. The cells were seeded on the microspheres at 1.5 × 10^4^ per well, upon which the plates were then incubated at 37°C in an atmosphere of 5% CO_2_. Dynamic cultures were carried out on a KS 260 control orbital shaker (IKA, Germany) with an orbital diameter of 10 mm. Both static and dynamic conditions were provided with slow agitation (50 rpm) for 5 min, prior to incubation, to ensure that there was even distribution of beads and cells. The dynamic culture plates were kept in an incubator under static conditions at 37°C and 5% CO_2_ for 24 h overnight, to allow cells to attach before plates were subjected to agitation. Cell culture was carried out for 13 days with readings taken on days 1, 3, 5, 7, 9 and 13. A fed-batch system was applied, with spent medium replaced every 48 h. Positive controls of 2D monolayer cultures of MG63s in Nunclon™ flat bottomed tissue culture 96-well microplates (Thermo Fisher Scientific, UK) were used to establish the relative cell attachment and growth on the microspheres.

The agitation speeds used for dynamic culture were pre-determined based on the Froude number (Fr). While this is generally attributed to large bioreactors, the geometry of a microwell in a 96-well plate allows a transfer of the principles to small scale. For this study, the Fr calculated were 0.66 and 0.70, which took into consideration any changes in volume of media due to evaporation, therefore impacting the liquid height. The values of the different variables used to quantify the Fr number are highlighted in Table 2. Using equation (4), a critical agitation rate around 340–350 rpm was identified as promoting out-of-phase mixing to give a suspension of microcarriers for effective cell expansion. Two agitation speeds were tested: 300 rpm, corresponding to the critical rate identified, and a lower rate 150 rpm to explore the hypothesis that increasing fluid shear forces may induce cell damage.

**Table 2.** Geometrical characteristics and calculated Fr_c_ and N_c_ (using minimum and maximum operating conditions) using 96-well microplates.

Variable		Value	Unit
Well inner diameter	d_i_	6.4 × 10^−3^	m
Orbital diameter	d_o_	1 × 10^−2^	m
Liquid volume (min)	V_min_	1.5 × 10^−7^	m^3^
Liquid volume (max)	V_max_	1.6 × 10^−7^	m^3^
Critical Froude number (min)	Fr_cmin_	0.65	
Critical Froude number (max)	Fr_cmax_	0.70	
Critical speed (min)	N_cmin_	340	min^−1^
Critical speed (max)	N_cmax_	350	min^−1^

Each dynamic experiment was carried out with a static control counterpart to provide the basis for a robust statistical analysis of the three conditions: static, 150 rpm and 300 rpm.

### Cell proliferation assay

Cell proliferation on each type of the microspheres in both static and dynamic conditions was measured using the Cell Counting Kit-8 (CCK-8) cell proliferation assay kit (Dojindo EU, Germany) following the supplier’s protocol. After incubation with the assay, the resulting solution was aspirated into Nunclon flat bottomed tissue culture 96-well microplates leaving behind the microspheres and cells due to the interference caused. At different points up to 13 days, optical densities were measured at a wavelength of 450–490 nm in a multiwall Safire2 plate reader (Tecan). All time points were assayed in triplicate with a mean and standard deviation calculated.

### Metabolite analysis

Offline glucose and lactate analyses were carried out using the YSI 2300 STAT bioanalyser (YSI Life Sciences, Yellow Springs, OH, USA) at days 1, 3, 5, 7, 9 and 13. Samples of 50 µL were taken and kept in 1 mL Eppendorf tubes. Each aliquot was kept in ice to ensure that the media did not reach a high temperature, preventing further metabolic activity or degradation. All samples were assayed in triplicates with a mean and standard deviation calculated.

### Confocal laser scanning microscopy imaging

Cell staining procedures were carried out to label the actin filaments of the cytoskeleton and the cell nucleus to assess morphologic appearance. Cells were first washed in situ in PBS, fixed with 4% paraformaldehyde (PFA) for 10 min at room temperature, washed twice further with PBS and consequently permeabilized with 0.5% Triton X-100 in PBS for 5 min at room temperature. The resulting cultures had the Triton X removed and were then stained with Acti-Stain™ 488 phalloidin (2.5 vol% phalloidin methanolic stock solution in phosphate-buffered saline) (Invitrogen, UK) for 20 min at room temperature in a dark chamber to reduce photobleaching and evaporation. Counter-staining with propidium iodide (PI) was carried out with the cell stained with a 1 µg/mL PI solution for 10 min also in dark conditions. Phalloidin and PI labelling were visualized under a Radiance 2100 confocal microscope (BioRad, Loughborough, UK).

### Statistical analysis

All statistical analyses were carried out using IBM SPSS Statistics version 22. First and foremost, the data sets were analysed using a normality test (Shapiro–Wilk test) revealing that specific data sets did not have a normal distribution. All normally distributed data were examined for equal variance using Levene’s statistics test, and a one-way analysis of variance (ANOVA) test carried out if the assumption of equal variance was valid. If the assumption of equal variance had been rejected, a robust tests of equality of means was carried out using the powerful Brown–Forsythe test. Depending on Levene’s test and the Brown–Forsythe test, a suitable post hoc analysis was carried out to establish the significance; tests used were Tukey’s honest significant difference (THSD) and Games–Howell test depending on whether the data showed equal variance or unequal variance, respectively. If the Shapiro–Wilk test indicated that the data was not normally distributed (p ⩽ 0.05), non-parametric tests were used, specifically the Kruskal–Wallis H test and Mann–Whitney U test, depending on the number of variables. Consequently, multiple pairwise comparisons were made using the Bonferroni correction method. The relevant statistical significance is illustrated on the graphical representations of the data which was produced in bar chart form.

## Results

### Cell proliferation of MG63 cells cultured on Ti-PGMs under static and dynamic conditions

**Figure 2.**
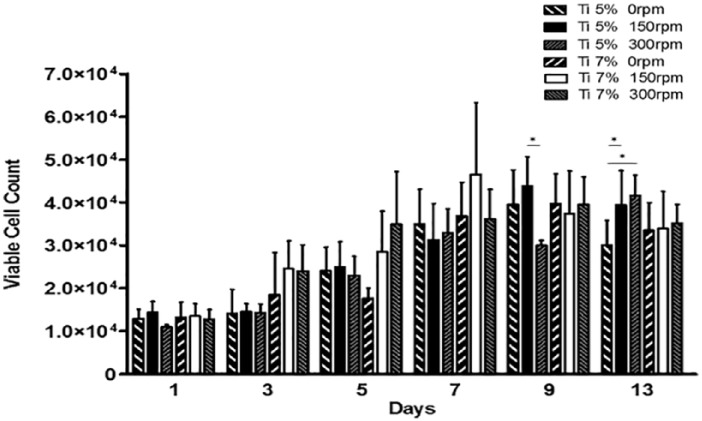
MG63 proliferation on Ti-PGMs in ultra-low attachment culture plates at days 0, 1, 3, 5, 7, 9 and 13 under static and dynamic conditions. Values represent mean ± SD (n = 3). * indicates p < 0.05. Ti: titanium dioxide.

Cell proliferation was assessed on two different Ti-PGMs compositions (5 and 7 mol%) and compared to control tissue culture plastic (TCP) 96-well microplates. Cell proliferation was assessed over the 13 days on the two different Ti-PGMs under the three different conditions 0, 150 and 300 rpm (Figure 2). Phosphate glass with 0 mol% TiO_2_ doping has not been included due to the excessive degradation of the material within 24 h of in vitro studies.^5^ The remaining compositions of phosphate glass promoted cellular growth from day 1 onwards. Glasses containing 5 mol% of TiO_2_ showed increases in viable cell numbers between day 1 and day 9 under static and 150 rpm conditions while with 300 rpm these increases were delayed until day 13. Cell proliferation on Ti7 showed similar growth profiles to Ti5. In comparison, cells cultured on TCP showed that under all agitation conditions, cells thrive early on with significant increases in cell numbers after only 1 day (*p < 0.05). When compared to the TCP surface, there is a stark difference with cell proliferation on Ti-PGMs. The cells cultured on TCP were significantly higher across all days when compared to the phosphate glass compositions on both static and 150 rpm (*p < 0.05) with 300 rpm showing the same trend (*p < 0.05). The maximum cell yield achieved under static conditions was highest with TCP compared to the phosphate glass materials; however, 150 rpm conditions provided the highest overall cell yield at a 4.8-fold increase in cell numbers at day 9 when compared to day 0. When analysing the maximum yields produced by the two different phosphate glasses, Ti7 produced its highest cell yield on day 7 with a 3.10-fold increase in cell number while Ti5 achieved a 2.9-fold increase on day 9 both occurring under 150 rpm conditions.

**Figure 3.**
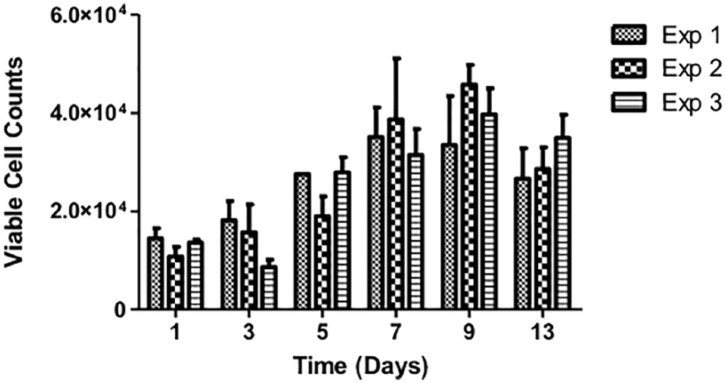
Growth profile of MG63 proliferation cultured on Ti 5 mol% under static conditions over 13 days where Exp 1, 2 and 3 denote three different static runs which were used as controls carried out in parallel with the dynamic studies (150 and 300 rpm). Exp 1: static only; Exp 2: static control for 150 rpm study; Exp 3: static control for 300 rpm study. Values represent mean ± SD (n = 3).

As these experiments were undertaken in batches, the consistency across experiments was determined (Figure 3). Static cultures using Ti-PGMs were used as controls during the 150 and 300 rpm studies; therefore, by showing that there are no significant differences among the three static cell proliferation profiles, it is possible to make comparisons across the different experiments.

### Impact of agitation rates on MG63 cell metabolism

**Figure 4.**
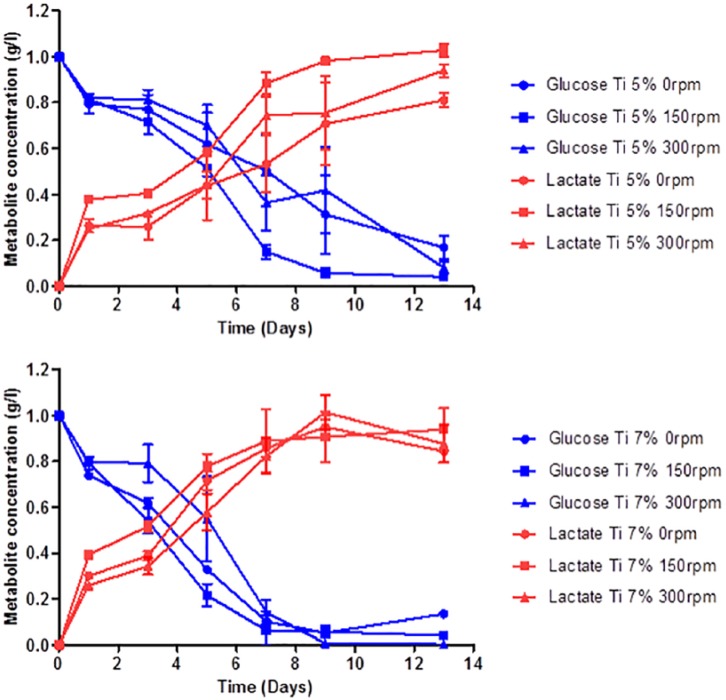
MG63 metabolic profiles for glucose consumption and lactate production as a function of time when cultured on Ti-PGMs at days 0, 1, 3, 5, 7, 9 and 13 under 0 rpm (•), 150 rpm (■) and 300 rpm (▲) condition. Values represent mean ± SD (n = 3). Ti: titanium dioxide.

Next, the metabolic activity of cells in culture was profiled (Figure 4). The profiles show that glucose consumption and consequent lactate production varied significantly under the different conditions investigated. The glucose profile for MG63 cells grown on Ti5 shows that more glucose was consumed by the cells in 96-well plates agitated under 150 rpm conditions compared to static conditions (*p < 0.05). The profiles for lactate production followed a similar trend in that cells cultured at 150 rpm produced significantly higher amounts of lactate through the course of 13 days compared to static conditions (*p < 0.05). By day 13, the MG63 cells agitated at 150 rpm produced more lactate than both static (***p < 0.001) and 300 rpm (*p < 0.05), with both dynamic conditions producing higher amounts of lactate than the MG63 cells in static culture. Surprisingly, cells cultured on phosphate glass doped with 7 mol% of TiO_2_ showed slight differences to metabolic activity of MG63 cells grown on Ti5 with more rapid glucose consumption occurring earlier on Ti7 than Ti5. Both static and 150 rpm conditions lead to greater glucose consumption compared to the 300 rpm condition at day 5 (*p < 0.05). Using the metabolite data, ratios of lactate production to glucose consumption (YLac/Glc) were calculated and revealed that cells cultured at 300 rpm (YLac/Glc = 1.33) exhibited higher conversion ratios than both 0 (YLac/Glc = 0.96) and 150 rpm (YLac/Glc = 0.97).

### Macroscopic evaluation of MG63 cell–Ti-PGMs clustering

**Figure 5.**
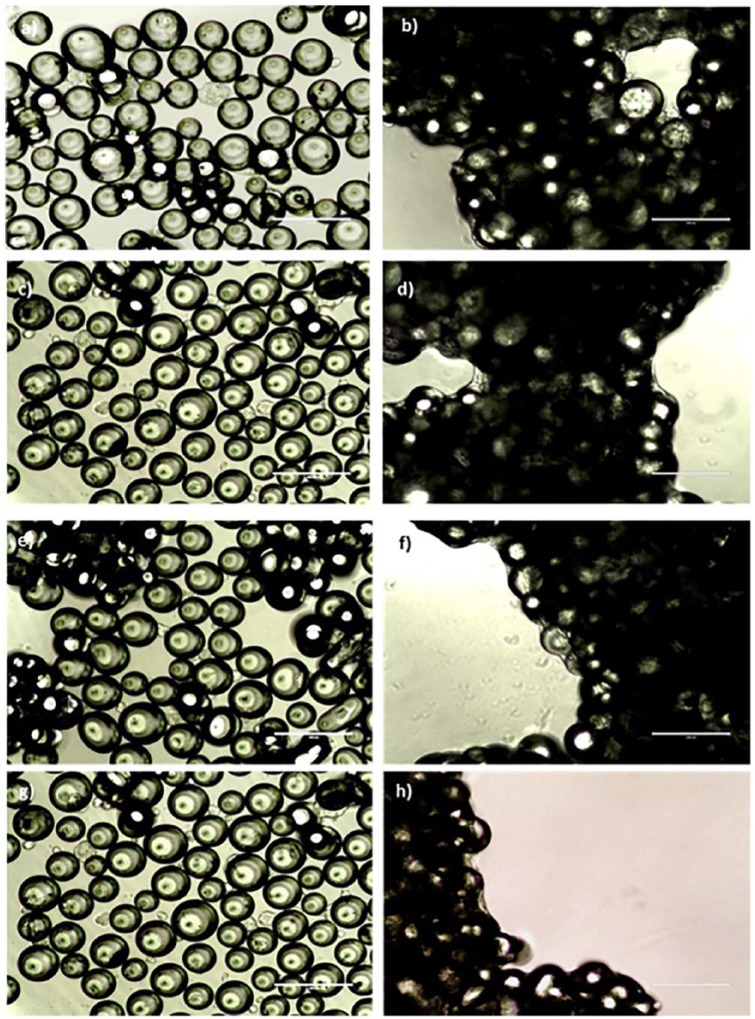
Formation of networks between MG63 cells and Ti-PGMs under different agitation rates. Phase contrast images illustrating MG63 cultured on Ti-PGMs over a 13 day period. Images of phosphate glasses doped with 5 mol% (a–d) and 7 mol% (e–h) were taken on day 1 (a, c, e and g) and day 13 (b, d, f and h) cultured under static (a, b, e and f) and 150 rpm (c, d, g and h) conditions. Images were taken under 20× magnifications where scale bars = 200 µm.

**Figure 6.**
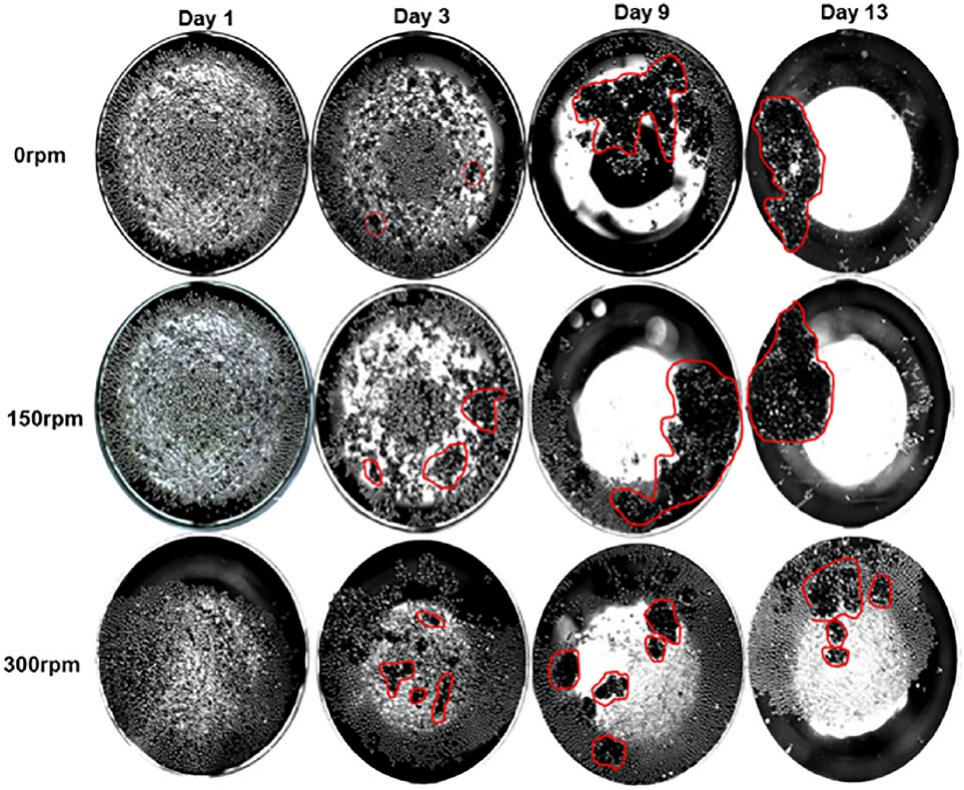
Formation of Ti-PGM-cell clusters under different agitation rates. Phase contrast images of an individual well illustrating MG63 cells cultured on Ti5 over a 13-day period using the same well from day 3 onwards. Red markings highlight areas of clustering. Images were taken under 2× magnification.

Cells were imaged over the course of the experiment using light microscopy. Figure 5 shows cell distribution throughout the well, including presence in spaces between adjacent microspheres in the monolayer. As the ultra-low attachment plates were maintained in static conditions for the first 24 h to allow settling of cells, there is no distinguishable difference between the day 1 images for any of the conditions, showing cells scattered evenly among the microspheres on both Ti5 and Ti7 materials. By day 13, cells have formed networks bridging between microspheres, consequently bringing them together and forming densely packed clusters. The darker areas seen in each of the images show densely populated clusters of microspheres and cellular material. The opaque nature of the images indicates a multi-layered structure consisting of at least two layers of microspheres. The way in which these structures form cannot be predicted, and fluid flow via orbital agitation is not a prerequisite given the static culture results. Macroscopic images were taken to show the extent of clustering of cell-microsphere material across the whole well (Figure 6). Although there were no statistically significant differences at day 1, at day 3 there are clear differences in the impact of orbital agitation on the formation of microsphere clusters. At day 3 under static conditions, there are a large amount of small microunits already formed, and these are on average smaller in size but larger in numbers compared to those clusters formed under 150 and 300 rpm. By day 9, the clusters formed in static and 150 rpm have developed into macrostructures and incorporate a vast quantity of microspheres initially seeded in the well. The structure in the static plate is less uniform than that visible in 150 rpm with large parts of the structure seeming to be in the early stages of integration into the main structure. The microunits formed in 300 rpm are comparably smaller than those produced under static and 150 rpm conditions. Furthermore, the quantity of clusters formed and number of microspheres remaining suggest that increasing the rate of agitation is limiting the ability of the clusters to incorporate adjacent microspheres, as illustrated by a large proportion of microspheres remaining unused by day 13.

#### Matrix deposition by MG63 cells on Ti-PGMs

**Figure 7.**
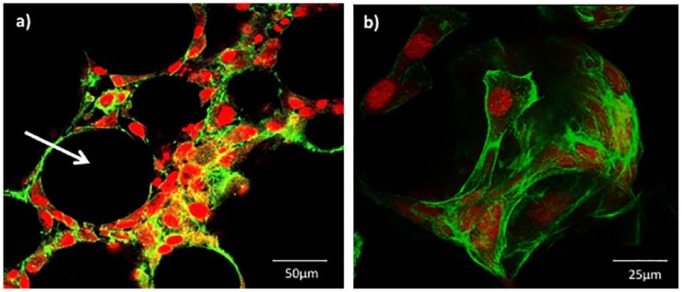
Confocal laser scanning microscopy images of MG63 cells attached to Ti5 glass microspheres prepared and stained on day 13 under 150 rpm conditions. Phalloidin stain was used to identify the actin filaments of the cytoskeleton (green) while the propidium iodide (PI) stain was used for the nuclear staining (red). Image (a) provides a sliced cross-section through a cluster of microspheres cultured with MG63 cells, while image (b) illustrates a 3D rendered image of an individual microsphere. Scale bar is (a) 50 µm and (b) 25 µm, respectively.

To further characterize MG63 cell interactions with TiO_2_ microspheres, cell–microcarrier interactions were documented using confocal laser scanning microscopy. Representative images show cells attached to Ti5 microspheres at day 13 post culture (Figure 7). Phalloidin staining revealed that the cell’s actin filaments aligned along the curved surface evenly covering the entire microsphere (Figure 7(b)). Processes extending from the edges of the cells aided the bridging between multiple microspheres, forming cell–microsphere aggregates. The spaces between microspheres were densely populated with MG63 cells (Figure 7(a)).

### Discussion

The present study examined the interaction between Ti-PGMs and MG63 cells under static and dynamic culture conditions on ultra-low attachment cell culture plates. The dynamic culture speeds studied were pre-determined using the dimensionless Froude number, which provided the critical agitation speed range of 340–350 rpm, based on measurements of the well geometry. The aim was to understand whether Ti-PGMs could be used in dynamic bioreactor environments to support the growth of osteoblastic cells to underpin the creation of tissue engineered bone. Within this study, we explored whether increasing the quantity of TiO_2_ within the microsphere would promote further proliferation of MG63 cells based on improved structural stability and surface properties. The results obtained from the study provide further insight into the biocompatibility of the Ti-PGMs and their application under dynamic culture conditions.

First, the MG63 cells were cultured on microspheres under static and dynamic conditions. From the three microsphere materials produced (Ti0, Ti5 and Ti7), the composition lacking Ti (Ti0) was found to be unsuitable for cell culture due to rapid degradation within 24 h, resulting in lack of structural integrity required to support cell attachment and growth. These observations are in agreement with those made by Abou Neel and Knowles^5^ who showed that phosphate glasses with <1 mol% TiO_2_ underwent rapid degradation and prevented MG63 cell growth. The remaining two microsphere types were assessed for their ability to support MG63 expansion, using TCP as a positive control. The results indicate that Ti5 and Ti7 provided support for cell attachment and growth, albeit to a lesser degree than the chemically enhanced TCP (Figure 3). These findings are consistent with those found previously in the lab for fibroblasts, where TCP exceeded the cell growth on the Ti-PGMs by approximately 50%.^7^ This is an expected outcome since the flat, delta-treated surface of TCP is optimized for cell attachment and proliferation. While the MG63 cells showed decreased proliferative capacity on Ti-PGMs compared to TCP, the substrates’ osteoinductive properties have not been matched by commercially available substrates; therefore, the trade-off in expanding fewer cells that have enhanced osteogenic properties can be more than favourable.^27^ Both Ti5 and Ti7 compositions promoted cell expansion and increased cell number over the 13-day culture period. There were no significant differences between the Ti5 and Ti7 microspheres in terms of overall cell proliferation, except for the days at which maximum cellular yield was achieved: Ti7 yielded higher cell numbers when subjected to 150 rpm conditions on day 7 as opposed to day 9 for Ti5 to reach their maximum yield. The limited differences in cell proliferation indicate the suitability of both materials as potential candidates for MG63 cell attachment and growth, and further quality markers of cell identity and function would need to be assessed to determine optimal substrate.

Confocal laser microscopy images illustrated the ability of the cells to wrap around and cover the curved surface of the microspheres (Figure 7(b)). It has been established in previous work carried out by Abou Neel and Knowles^5^ that partial release of PO_4_^3^^−^ ions from phosphate glasses doped with 5 mol% TiO_2_ took place over 13 days when in a solubilizing solution. However, there were no differences between Ti5 and Ti7 in terms of cell viability and illustrated by the densely packed cells seen in confocal images (Figure 7(a)) suggesting that the local microenvironment in terms of ion release was similar between the two compositions.

Studying the effect of dynamic culture conditions on cell proliferation provides crucial insight into the applicability of the material as a substrate for use in scalable bioreactors, and the effect that mixing has on cell proliferation. The use of the Froude scaling law^15^ provided a foundation for identifying which orbital agitation speeds should be studied to understand the impact of mixing patterns upon cell proliferation. This was done because applying a model that defines critical orbital agitation speed (at which the contents of a cylindrical vessel are thoroughly mixed, and the microspheres contained within are theoretically in suspension) should predict effective cell expansion due to homogeneous nutrient and oxygen levels. The effect of dynamic culture conditions upon cell numbers is documented in Table 3, which indicated that the highest cell yields could be achieved by 150 rpm agitation, with values of 2.9- and 3.1-fold increases when cells are cultured on Ti-PGMs compared to values ranging from 2.64- to 2.77-fold increase for 0 and 300 rpm. However, the highest agitation rate, 300 rpm, on average produced the lowest yield increase across all three different materials studied, which brings into question the applicability of the model developed by Weheliye et al.^15^ This model is limited in that it fails to factor in the density of any particulates higher in density than the fluid within the vessel. The densities of the materials used in the study are 2.65 and 2.71 g/cm^3^ for Ti5 and Ti7, respectively, higher than commercially available microcarriers whose densities are similar to that of water (1 g/cm^3^). At 150 rpm, mixing would have occurred above the microspheres in the two toroidal vortices, while at 300 rpm, the vortices would have been closer to the surface. We also hypothesize that increasing agitation rates from 150 to 300 rpm could have a detrimental effect on cell function due to higher fluid shear forces experienced by the cells.

**Table 3.** Fold increase values indicating the maximum cells yield (time point) achieved over the course of the experiment under the different conditions and materials used.

Material	Condition (day)		
	0 rpm	150 rpm	300 rpm
Ti5	2.64 (9)	2.92 (9)	2.77 (13)
Ti7	2.65 (9)	3.10 (7)	2.63 (9)
TCP	4.59 (13)	4.80 (9)	4.29 (13)

TCP: tissue culture plastic.

Values rounded to 2 s.f.

We also aimed to understand how dynamic cell culture conditions affected the consumption of feed substrates and their conversion to waste products within the whole well by the cells. The glucose and lactate concentrations were monitored to indirectly evaluate cell viability. Glucose acts as a primary energy substrate for mammalian cells to produce ATP through oxidative phosphorylation or via anaerobic glycolysis.^28^ The values produced by the YSI Bioanalyser provide an overall average of the metabolite concentration in the well. Therefore, to fully grasp how effectively each condition and material can support cell metabolism, it is important to normalize these values to viable cell number. As media changes were made every 48 h, which were carried out after each cell proliferation assay time point, calculating the corresponding glucose consumptions for each current time point was possible and subsequently these values were normalized to viable cell number.

The ratio of lactate production to glucose consumption per cell (YLac/Glc) indicates the metabolic pathway the cells prefer to use as a mode of energy production.^28,29^ Schop et al. stated that an YLac/Glc value of 2 indicates that the cells inefficiently convert glucose using the glycolytic pathway as opposed to oxidative phosphorylation to produce energy. The ratio indicates that 2 moles of lactate, the maximum yield possible, are created per mole of glucose. A lower ratio indicates that glucose consumption generates fewer units of lactate in a more energy-efficient process.^28^ Cells cultured under 300 rpm (YLac/Glc = 1.33) showed higher conversion ratios than both 0 rpm (YLac/Glc = 0.96) and 150 rpm (YLac/Glc = 0.97), consistent with studies carried out by Schop et al.,^10^ who analysed metabolic activity of mesenchymal stem cells on microcarriers. The values show that 300 rpm produced the least favourable conditions among those studied, correlating with lower cell yields. This indicates that the higher agitation condition was not ideal for the cells because they were less efficient at metabolizing glucose.

Fluid flow-induced shear stress can contribute to metabolic quiescence in some cell lines via the action of transcription factors,^30^ which potentially explains why a higher YLac/Glc ratio was produced at 300 rpm and also why cell expansion was not as high at 300 rpm compared to 150 rpm. It has been well documented that there are significantly lower oxygen transfer rates in 96-well microplates, which can potentially induce anaerobic conditions. However, it is unlikely that this would influence the results because each condition carried the same volume of media, with little variation across ultra-low attachment and surface-treated TCP.^31^ Given the application of agitation providing no observable benefit in cell metabolism compared to static culture, the argument of whether the applying agitation when culturing on doped phosphate glass is beneficial arises. It is hard to conclude whether the unfavourable shift in metabolic pathway is attributed to the shear stress caused by fluid flow, or potentially down to mass transfer limitation into the core of the structure.

The MG63 cells are very robust in nature and have a tendency to show high proliferation rates.^32^ Therefore, while the cells have given an early indication to the ability of the material to support cell growth under both static and dynamic conditions on both types of phosphate glass studied, the cells’ ability to withstand comparatively higher shear and their documented growth kinetics provide only primary understanding of how effective the material can be. Further studies are needed with more immature cells such as MSCs, which are also more clinically relevant, to establish the full extent that fluid dynamics impact cell proliferation and differentiation when cells are cultured on these doped phosphate microspheres.
